# Static and Dynamic Foot Pressure Changes Among Diabetic Patients With and Without Neuropathy: A Comparative Cross-Sectional Study

**DOI:** 10.7759/cureus.45338

**Published:** 2023-09-16

**Authors:** Manjunath Totaganti, Ravi Kant, Raj Kumar Yadav, Meenakshi Khapre

**Affiliations:** 1 Internal Medicine, All India Institute of Medical Sciences, Rishikesh, IND; 2 General Medicine, All India Institute of Medical Sciences, Rishikesh, IND; 3 Nursing, All India Institute of Medical Sciences, Rishikesh, IND; 4 Physical Medicine and Rehabilitation, All India Institute of Medical Sciences, Rishikesh, IND; 5 Social Preventive Medicine, All India Institute of Medical Sciences, Rishikesh, IND

**Keywords:** glycemic control, neuropathy, diabetic foot, foot pressure mapping, diabetes mellitus management

## Abstract

Introduction: Foot ulceration is a frequent diabetic complication with potentially fatal consequences. The pathophysiology of neuropathic ulcers in the diabetic foot is thought to be influenced by abnormal plantar pressures.

Aim: This study aimed to compare the maximum peak pressures among diabetic patients with and without neuropathy. The secondary aim was to evaluate the effect of glycemic control on pressure changes in both feet.

Materials and methods: The study used 62 diabetic individuals as participants. BMI was calculated, as well as illness duration, hemoglobin A1c, and the existence of neuropathy. Plantar pressure was measured in static (standing) and dynamic (walking/taking a step on the mat) settings for all patients using the BTS P-Walk system. The plantar pressures (kPa) at the five metatarsal regions, the midfoot region, and the medial and lateral heel regions were measured.

Results: We found that the dynamic maximum pressures were significantly higher in patients with diabetic neuropathy (DN) compared to diabetics without neuropathy at the first metatarsal and mid-foot area in both feet (p<0.05). We also found significantly elevated plantar pressure in patients with poor glycemic control under the second metatarsal head in the right foot (p<0.05).

Conclusion: Persons with DN have higher maximum plantar pressures compared to diabetics without neuropathy. Patients with poor glycemic control also have a higher maximum pressure.

## Introduction

Diabetes is the primary health concern in the current era, posing a significant burden on health and social and economic development. According to a systematic study of the Global Burden of Disease Study 2021, the global diabetes population in 2021 was 529 million, with a worldwide age-standardized total diabetes prevalence of 6.1% (5.8-6.5) [1]. Distal symmetric polyneuropathy (DSPN) is the most common type of diabetic peripheral neuropathy. It constitutes about 75% of cases [2]. A study showed that 50% of diabetic patients have diabetic neuropathy (DN) [3]. DSPN is the major cause of morbidity and disability; unrecognized neuropathy leads to foot ulceration and amputation. Studies showed an increased risk of developing ulcers up to seven times, 15% of patients developed osteomyelitis at or after the time of diagnosis, and 15.6% required amputation [4]. Higher plantar pressure has been linked to ulcer formation in diabetics in the past [5]. It is well-recognized that increased pressure in diabetic individuals with peripheral neuropathy may cause foot ulceration [6]. Currently, there is no data on quantifying foot pressure on screening and prevention. There are limited data on pressure changes among diabetic patients in India. Given the high prevalence of diabetes in India and Southeast Asia, this knowledge might influence policies and practices. As a result, this study was carried out to better understand the changes in plantar pressure between type 2 diabetes mellitus (T2DM) with and without DSPN.

## Materials and methods

This study was a 15-month cross-sectional study conducted at a tertiary-care teaching hospital in the division of diabetes and metabolism (internal medicine) in collaboration with the physical medicine and rehabilitation department. After obtaining institutional ethics committee clearance (All India Institute of Medical Sciences, approval number AIIMS/IEC/20/502), 150 T2DM patients were screened after taking written and informed consent. We included diabetic patients aged more than 18 years of both sexes who can walk independently without any visible, apparent gait abnormalities. Patients with an abnormal gait, non-healing chronic ulcers, acute ulcers, amputation of one or both limbs, foot deformity, spine deformity, severe cardiovascular disease (New York Heart Association class 3 and 4), nephropathy (chronic kidney disease, stage 4 and 5), and other illnesses that impair quality of life and movement were all excluded. We also excluded pregnant women and patients who were unable to follow commands.

All participants underwent assessment including history, the duration of diabetes, and any diabetic complications. Height, weight, and BMI were also calculated. They were evaluated for nephropathy by 24-hour urine proteins and retinopathy by a fundus camera. Neuropathy assessment was done using the neuropathy analyzer VIBROTHERM Dx (Diabetik Foot Care India Pvt Ltd, Chennai, India), including vibration perception threshold by a biothesiometer (Vibrotest, Bruel and Kjaer, Nærum, Denmark), hot and cold perception threshold, and 10 g Semmes-Weinstein monofilament test (Diabetik Foot Care India Pvt Ltd, Chennai, India). The normal values are shown in Table 1.

**Table 1:** The normal values of the neuropathy test

Test	Normal
Hot perception threshold	≤42°C in all areas
Cold perception threshold	≥20°C in all areas
Vibration perception threshold	≤15 volts in all areas
10 g Semmes-Weinstein monofilament	Able to perceive >4 areas out of 6 spots

Each foot was examined in the six regions: great toe, first metatarsal head, third metatarsal head, fifth metatarsal head, midfoot, and heel. The sensitivity and specificity of neuropathy identification differ between studies. However, Mythili et al. discovered that the monofilament study's sensitivity and specificity were 98.5 % and 55 %, respectively. She also discovered that the vibration perception threshold's sensitivity and specificity were 86% and 96%, respectively [7].

The diagnosis of DN was made if any one of the tests mentioned in Table 1 were found to be abnormal. Those patients who had all the tests normal were considered diabetic patients without neuropathy. Based on these results, they were grouped as diabetic control (DC) and DN.

**Figure 1:**
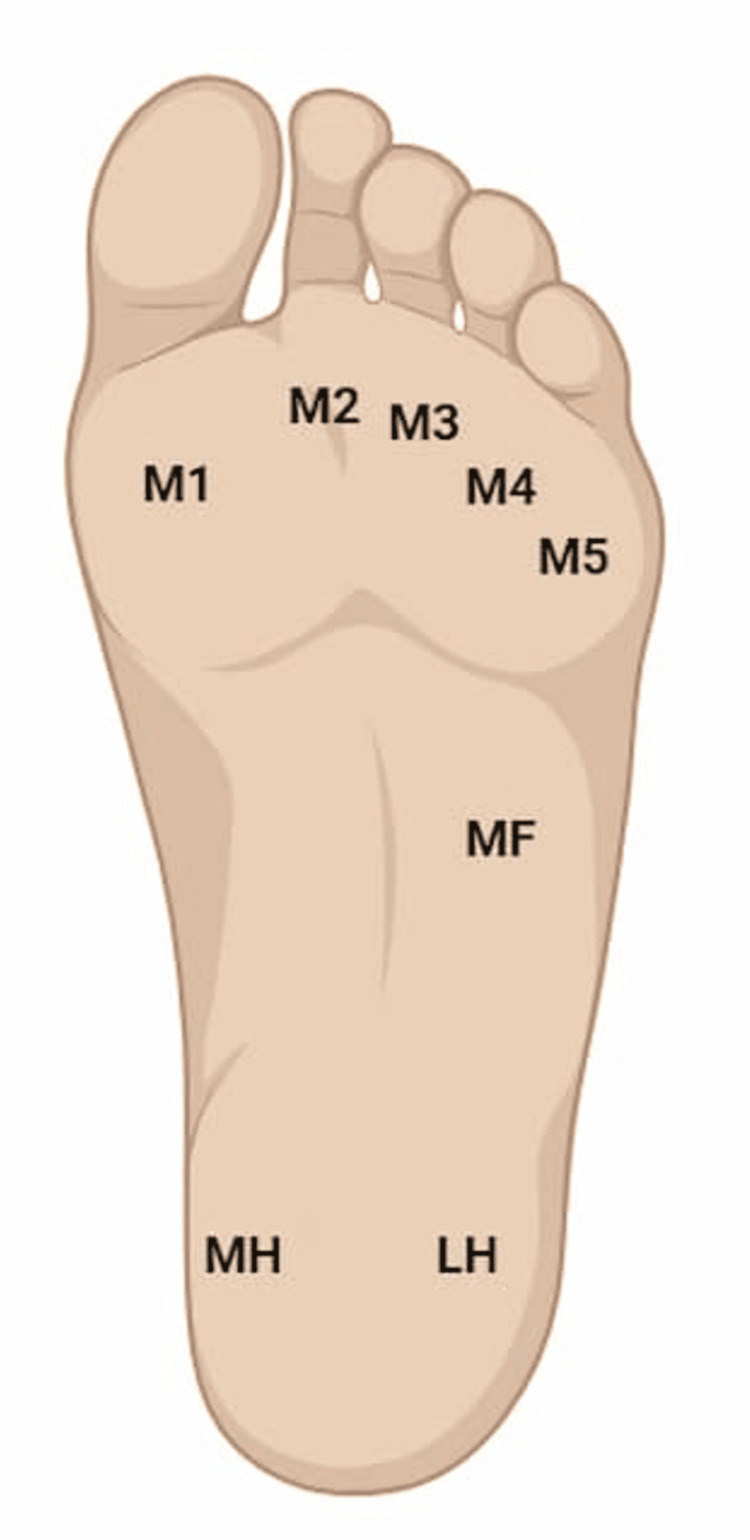
The areas of the foot at which the pressures were recorded M1: first metatarsal area, M2: second metatarsal area, M3: third metatarsal area, M4: fourth metatarsal area, M5: fifth metatarsal area, MF: midfoot area, MH: medial heel, LH: lateral heel Created with BioRender.com

Plantar pressure assessment was done using the BTS P-Walk system. The maximum pressures were taken in standing (static) and walking across the mat system (dynamic). Standardized instructions were given to participants. The pressures were documented in the first metatarsal area (M1), the second metatarsal area (M2), the third metatarsal area (M3), the fourth metatarsal area (M4), the fifth metatarsal area (M5), the midfoot area (MF), the medial heel area (MH), and the lateral heal area (LH), as shown in Figure 1.

Data were recorded using Microsoft Office Excel (Microsoft, Washington, USA) and analyzed using SPSS Statistics version 25 (IBM Corp. Released 2017. IBM SPSS Statistics for Windows, Version 25.0. Armonk, NY: IBM Corp.). Descriptive statistics were employed to summarize various clinical and demographic parameters. Categorical variables were presented as frequencies and proportions, while continuous variables were reported as either mean ± standard deviation or median with interquartile range, depending on the data distribution.

To assess the significance of pressure changes between the two groups, the Mann-Whitney U test was applied. A p-value of less than 0.05 was considered statistically significant.

## Results

Based on the abovementioned test results, the subjects were grouped according to the presence or absence of neuropathy: DN, in which 31 patients with T2DM were found to have neuropathy based on one positive test (Table 1), and DC, in which 31 patients with T2DM were found to have no neuropathy (those who had all the tests in Table 1 were normal).

Demographical data

The mean age in the DN and DC groups were 50.7 ± 10.3 and 47.5+8.6, respectively. The study groups included 18 male patients (58%) and 13 female patients (41.9%) in each group. The mean BMI in the DN group was 24.9 ± 4.1 and in DC was 26.0 ± 3.4. On comparing the age and BMI, there was no statistically significant difference between them (p=0.13 and 0.1, respectively). These data are compiled in Table 2.

**Table 2:** Baseline demographic data in both groups SD: standard deviation, BMI: body mass index, DN: diabetic neuropathy, DC: diabetic control

	DN group		DC group		
	Mean	SD	Mean	SD	p-value
Age (years)	50.29	10.329	47.58	8.682	0.13
Height (cm)	160.58	7.027	158.42	10.363	0.72
Weight (kg)	64.58	12.976	65.74	12.775	0.22
BMI (kg/m^2^)	24.96	4.18	26.06	3.46	0.1
Duration of diabetes	6.66	3.76	4.45	2.66	0.009

The mean glycated hemoglobin (HbA1c%) in the DN group was 8.6 ± 1.6 and in the DC group 8.4 ± 2.2, without statistically significant difference. The duration of diabetes was significantly higher in the DN group compared to the DC group (6.6 ± 3.7 years vs 4.4 ± 2.6 years; p=0.009). This is depicted in Figure 1. The v baseline investigations are compared among both groups, as shown in Table 3.

**Table 3:** Baseline investigations among the two groups SD: standard deviation, HbA1c: hemoglobin A1C, HDL: high-density lipoprotein, LDL: low-density lipoprotein, DN: diabetic neuropathy, DC: diabetic control

	DN group			DC group		
	Mean		SD	Mean	SD	p-value
Haemoglobin (gm/dl)	12.539	1.8805		13.161	1.8708	0.19
Total leucocyte count (cells/microlitre)	6988.16	1947.901		6810.55	2036.048	0.72
HbA1c (%)	8.629	1.6387		8.403	2.291	0.65
Fasting glucose (mg/dl)	168.65	63.283		155.61	63.439	0.42
Total cholesterol (mg/dl)	182.45	43.638		165.65	57.010	0.49
Serum triglycerides (mg/dl)	150.29	62.786		146.52	67.217	0.26
HDL (mg/dl)	47.13	10.661		46.35	6.306	0.65
LDL (mg/dl)	101.58	27.449		91.77	35.23	0.42
24-hour urine protein (mg/24hr)	278.71	229.490		167.03	112.368	0.01

Plantar pressure (KPa) comparison between the two groups

**Figure 2:**
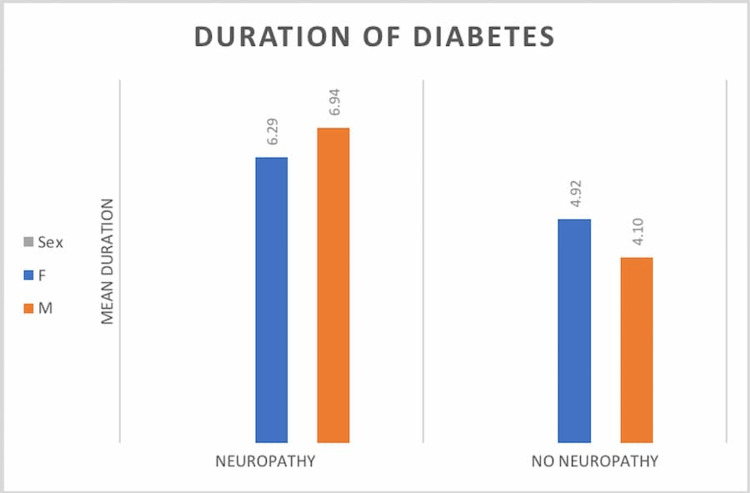
Duration of diabetes among the two groups (in years)

The maximum pressures in left static pressures were higher in DN in M1, M2, M3, M4, and MH, although the difference was not statistically significant. The maximum pressures in right static pressures were higher in DN in M2 and M3, although the difference was not statistically significant. The data is tabulated in Table 4 and the graphical representation is in Figure 2.

**Table 4:** Maximum static pressures in the left and right foot among groups IQR: interquartile range, M1: first metatarsal area, M2: second metatarsal area, M3: third metatarsal area, M4: fourth metatarsal area, M5: fifth metatarsal area, MF: midfoot area, MH: medial heel, LH: lateral heel

Left static	DN group (n=31)		DC group (n=31)		
Maximum pressure (in kilopascal)	Median	IQR	Median	IQR	p-value
M1	69	83-33	55	69-17	0.10
M2	84	120-54	72	104-30	0.25
M3	93	120-93	72	113-37	0.34
M4	86	114-43	74	102-40	0.55
M5	56	74-35	61	75-30	0.89
MF	60	81-43	72	98-26	0.34
MH	182	220-132	165	203-132	0.53
LH	150	207-118	154	197-131	0.83
Right static (in kilopascal)					
M1	53	92-31	55	83-27	0.87
M2	93	128-38	83	112-46	0.53
M3	97	144-52	94	120-52	0.50
M4	83	136-54	85	117-50	0.63
M5	59	87-38	69	85-47	0.48
MF	39	55-12	47	71-29	0.24
MH	138	190-119	138	197-99	0.75
LH	123	156-102	148	176-95	0.33

**Figure 3:**
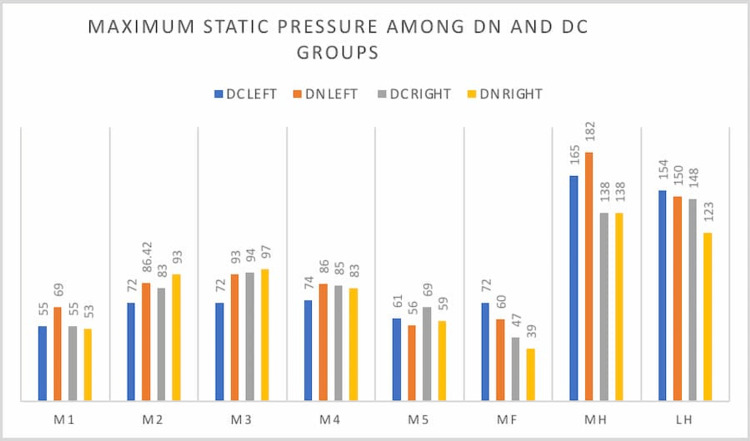
Maximum static pressure among DN and DC groups in both foot DN: diabetic neuropathy, DC: diabetic control, M1: first metatarsal area, M2: second metatarsal area, M3: third metatarsal area, M4: fourth metatarsal area, M5: fifth metatarsal area, MF: midfoot area, MH: medial heel, LH: lateral heel

The maximum dynamic pressure in the left foot was significantly higher in the DN group at the first metatarsal head (p-value 0.023) and the midfoot (p-value 0.014), and in the right foot, it was significantly higher in the DN group at the midfoot (p-value 0.031). The rest of the values are shown in Table 5 and Figure 3.

**Table 5:** Maximum dynamic pressures in the left and right foot among the groups IQR: interquartile range, M1: first metatarsal area, M2: second metatarsal area, M3: third metatarsal area, M4: fourth metatarsal area, M5: fifth metatarsal area, MF: midfoot area, MH: medial heel, LH: lateral heel

Left dynamic	DN group (n=31)		DC group (n=31)		
Maximum pressure (in kilopascal)	Median	IQR	Median	IQR	p-value
M1	627	809-512	474	660-352	0.023
M2	748	867-669	734	893-643	0.95
M3	772	885-632	773	913-656	0.58
M4	725	777-595	637	783-526	0.69
M5	505	671-363	462	561-352	0.23
MF	218	383-92	116	206-76	0.014
MH	563	688-463	600	755-507	0.33
LH	543	626-457	545	598-444	0.88
Right dynamic (in kilopascal)					
M1	266	310-173	169	228-109	0.002
M2	391	424-330	354	430-254	0.71
M3	377	432-332	376	449-305	0.93
M4	323	409-257	290	361-217	0.19
M5	240	264-153	172	221-115	0.07
MF	99	146-33	44	115-24	0.023
MH	282	354-233	279	318-214	0.72
LH	136	211-54	167	236-139	0.25

**Figure 4:**
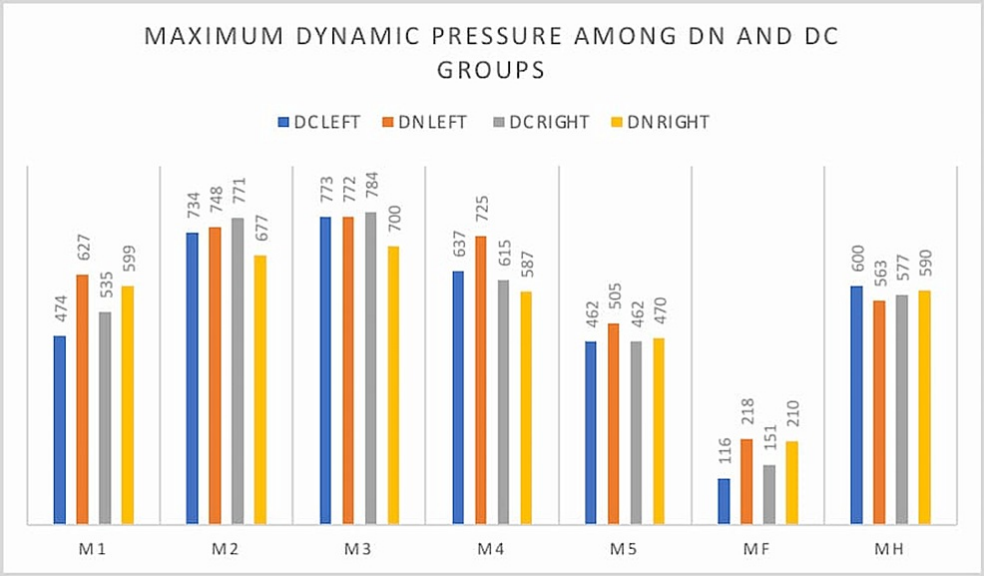
Maximum dynamic pressure among both groups DN: diabetic neuropathy, DC: diabetic control, M1: first metatarsal area, M2: second metatarsal area, M3: third metatarsal area, M4: fourth metatarsal area, M5: fifth metatarsal area, MF: midfoot area, MH: medial heel

**Figure 5:**
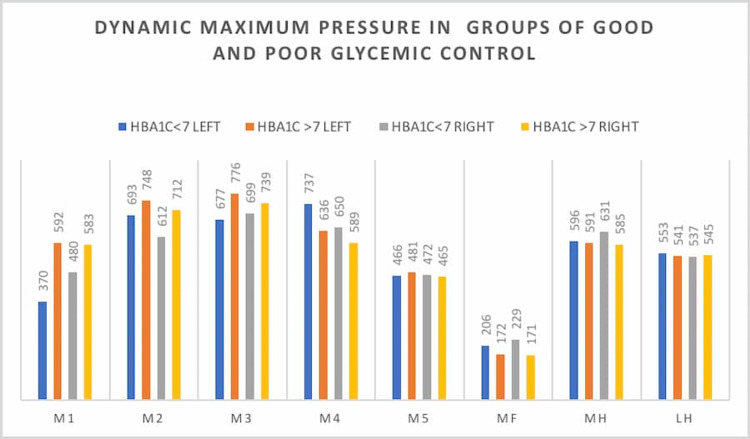
Dynamic maximum pressure in groups of good and poor glycemic control HbA1C: hemoglobin A1C, M1: first metatarsal area, M2: second metatarsal area, M3: third metatarsal area, M4: fourth metatarsal area, M5: fifth metatarsal area, MF: midfoot area, MH: medial heel, LH: lateral heel

We also grouped the patients based on the HbA1c value as good and poor glycemic control. We compared the dynamic pressures in both groups (Table 6 and Figures 4-5) and found them to have statistically significant higher pressure in poor glycemic control at the M2 area in the right foot (p-value 0.019).

**Table 6:** Right and left dynamic pressures among the patients with good and poor glycemic control (N=62) IQR: interquartile range, HbA1c: hemoglobin A1C, M1: first metatarsal area, M2: second metatarsal area, M3: third metatarsal area, M4: fourth metatarsal area, M5: fifth metatarsal area, MF: midfoot area, MH: medial heel, LH: lateral heel

	HbA1c <7 (n=9)		HbA1c >7 (n=53)		
	Median	IQR	Median	IQR	p-value
Left dynamic pressure (in kilopascal)					
M1	370.00	677-278.5	592.00	713.5-442	0.22
M2	693.00	768.5-465.5	748.00	888.5-669	0.065
M3	677.00	816-578	776.00	896-656	0.20
M4	737.00	795-523.5	636.00	767.5-561	0.50
M5	466.00	881.5-397	481.00	617.5-347.5	0.30
MF	206.00	319-114.5	172.00	270-82.5	0.47
MH	596.00	756.5-558	591.00	721-471.5	0.56
LH	553.00	587-519	541.00	632-450	0.78
	Median	IQR	Median	IQR	p-value
Right dynamic pressure (in kilopascal)					
M1	480.00	680.5-419.5	583.00	728-453	0.35
M2	612.00	691.5-581	712.00	930-648	0.019
M3	699.00	775-599	739.00	910.5-649	0.13
M4	650.00	655-527	589.00	755.5-531.5	0.88
M5	472.00	545.5-419	465.00	612.5-379	0.96
MF	229.00	305-145	171.00	286.5-88.5	0.37
MH	631.00	672.5-480.5	585.00	728-459	0.71
LH	537.00	656-444.5	545.00	678-404	0.99

## Discussion

The direct or indirect consequences of hyperglycemia on the vascular system are the leading cause of morbidity and death in both T1DM and T2DM [8]. Diabetes is a metabolic disorder that can lead to multiple complications; one such complication is a diabetic foot ulcer. Diabetes can lead to morbidity and mortality. A diabetic's lifetime risk of having a foot ulcer is estimated to be as high as 25% [9]. The pathogenesis of neuropathy is not fully understood. The potential mechanism of neuropathy involves a convergence of factors. The metabolic repercussions of chronic hyperglycemia result in direct axonal damage, while microvascular dysfunction induces anoxic injury to nerves [10]. Furthermore, oxidative stress [11], the deleterious impact of glycated products, and the activation of the polyol pathway contribute to this process.

A widely recognized risk factor is plantar pressure, which has been demonstrated to be higher in individuals with diabetes compared to those without diabetes. This heightened plantar pressure has been linked to the development of ulcers, as evidenced by findings from a prospective study [12].

The maximum dynamic pressure in the DN group was considerably greater than in the DC group, even though the groups were age, sex, and BMI matched. The diabetic duration was significantly higher in the DN group. This was comparable with similar results found by Sacco et al., who demonstrated higher plantar pressure among various stages of neuropathy patients compared with DC in the heel and forefoot areas [13]. The authors further attributed the increased pressures to small muscle atrophy, joint deformities, and sensory abnormalities [13].

Richard et al. demonstrated higher dynamic pressures in the medial and lateral metatarsals [14]. In a study by Bacarin et al., midfoot peak plantar pressure and pressure-time integrals in overall plantar areas were significantly higher in diabetic neuropathic subjects. The authors attributed this to neuropathic subjects' loss of protective sensation, which can lead to compensatory musculoskeletal mechanisms that alter the foot rollover mechanism [15].

in this study population, we compared the foot pressure changes in patients with poor and good glycemic control indicated by glycated hemoglobin concentration, although glycated hemoglobin concentration was considerably higher in both diabetes groups and was comparable in both groups. The maximum pressure while walking was considerably higher in patients with poor glycemic control at toe 1 and first, second, and third metatarsal heads. At the right second metatarsal head, there was a statistically significant difference.

Similar results were found by Qiu et al. They found that HbA1c was positively correlated to the maximum pressure at the first metatarsal head [15]. Some studies, such as those by Ahroni et al. [16], Halawa et al. [17], and Qui et al. [18], found no link between HbA1c and maximal plantar pressures in diabetic patients. A cross-sectional study between diabetes and non-diabetic Chinese patients found that the fasting blood sugars were significantly higher in the former group [17].

These findings, combined with previous results, suggest that the link between glycemic changes and peak plantar pressure is hazy and that it is more likely to be exerted indirectly through the effect of glycemia on the natural progression of peripheral neuropathy and the resulting impact of neuropathy on plantar pressure changes, foot abnormalities, and gait changes. The results of our study need to be explored by future studies on the Indian population.

Attempts to establish a maximum pressure threshold for ulceration have been unsuccessful, and the apparent magnitude of pressure levels across studies is variable [18]. Different studies across the world have different thresholds. Veves et al. discovered that ulceration requires a pressure of over 1000 kPa when walking barefoot [15]. Armstrong et al. examined peak pressure in 219 diabetic individuals and indicated that 700 kPa is the ulceration threshold in a case-control study [19]. Understanding and avoiding neuropathic ulcers is becoming more critical as the number of people with diabetes rises. Identification of these pressures has screening value in identifying the area of high risk. Casting, insoles, rocker shoes, and tailored shoes are among the clinically utilized ways to reduce pressure when walking for persons with diabetic neuropathies. These devices function by increasing the area of weight-bearing force and preventing localized pressure on the foot.

Limitations of the study

Observational studies rely on the selection of participants from a specific population or sample, which is known as selection bias. It may be challenging to obtain a representative sample during the COVID-19 pandemic due to limitations on patient visits, lower participation rates, or adjustments in healthcare-seeking behavior. The absence of a comparison group of healthy individuals to know the pressure changes variation in diabetic patients without neuropathy. The study misses an opportunity to gather vital data regarding the development of the disease by failing to follow individuals who develop ulcers after having increased foot pressures. Understanding the progression of ulcer formation over time, its risk factors, and relevant therapies might be aided by this data. The ability to demonstrate a causal link between higher foot pressures and ulcer formation is constrained by the lack of follow-up data. Without monitoring the development of ulcers in patients with high pressures over time, it is difficult to say if the pressures alone cause ulceration or if other variables play a role.

## Conclusions

In conclusion, persons with DN have higher maximum plantar pressures. Poor glycemic control indicated by higher glycated hemoglobin may also correlate to higher pressures in those patients. Future research is needed to fully understand the processes and effects of neuropathy and glycemic management on diabetic foot pressure variations.
